# Long-term care insurance in China: Current challenges and recommendations

**DOI:** 10.7189/jogh.14.03015

**Published:** 2024-09-27

**Authors:** Simiao Chen, Linye Li, Lirui Jiao, Sen Gong, Zhuoran Wang, Haitao Liu, Pascal Geldsetzer, Juntao Yang, Till Barnighausen, Chen Wang

**Affiliations:** 1 Chinese Academy of Medical Sciences and Peking Union Medical College, Beijing, China; 2 Heidelberg Institute of Global Health (HIGH), Faculty of Medicine and University Hospital, Heidelberg University, Heidelberg, Germany; 3 Chinese Academy of Social Sciences, Beijing, China; 4 Department of Health Policy and Management, Gillings School of Global Public Health, University of North Carolina at Chapel Hill, Chapel Hill, North Carolina, USA; 5 Center for International Knowledge on Development, Beijing, China; 6 Division of Primary Care and Population Health, Department of Medicine, Stanford University, California, USA; 7 State Key Laboratory of Medical Molecular Biology, Institute of Basic Medical Sciences, Chinese Academy of Medical Sciences and Peking Union Medical College, Beijing, China; 8 Department of Global Health and Population, Harvard School of Public Health, Boston, Massachusetts, USA; 9 National Clinical Research Centre for Respiratory Diseases, Beijing, China; 10 Department of Pulmonary and Critical Care Medicine, Centre of Respiratory Medicine, China-Japan Friendship Hospital, Beijing, China

In establishing long-term care insurance (LTCI), China has recognised the need for a robust and sustainable health care and insurance system to effectively meet the evolving needs of its ageing population and the corresponding increasing demand for long-term care (LTC) services. In 2016, China introduced a pilot LTCI program in 15 cities [1]. By 2020, China had expanded the program to 20 further cities in two key pilot provinces (Shandong and Jilin), as well as to an additional 14 cities and areas in other provinces [2]. Since its inception, efforts have also been made to improve the pilot program’s coverage and benefits. However, there are still many challenges that need to be resolved in the process of constructing a national LTCI system. China has not reached a unified consensus on a financing scheme for LTCI nor on the scope of insurance coverage, and significant gaps remain in the required quantity and quality of technology and personnel for LTC service provision.

With this context as background, in this viewpoint, we briefly compared China’s LTCI implementation experience with those of other countries. We analysed the environment for LTCI in China and provided recommendations for the conceptualisation and management of China’s LTCI system.

## INTERNATIONAL COMPARISON OF LTCI SYSTEMS IN CHINA AND OTHER COUNTRIES

A number of countries have undergone population ageing earlier than China and have therefore taken the initiative to form relatively comprehensive LTCI systems to meet their ever-increasing LTC needs. Prevalent practices in these countries include introducing legislation and regulations to support LTCI, promoting home and community-based services (HCBS), and providing partial government funding for LTCI.

We summarised relevant legislative and regulatory initiatives undertaken by several high-income countries and China to promote the development of their LTCI systems. These initiatives support LTCI by guaranteeing providers. Country legislative and regulatory approaches differ, to an extent, according to whether insurance is provided via the welfare state (UK), social insurance (China, Germany, Japan, and South Korea), or commercial insurance (USA) (**Table 1**).

**Table 1:** Comparison of LTCI systems in selected countries

	UK [3]	China [4,5]	Germany [6,7]	Japan [8,9]	South Korea [10]	USA [11]
**Financial protection model**	Welfare state	Social insurance	Social insurance	Social insurance	Social insurance	Commercial insurance
**Legislation**	Caring for people: community care in the next decade and beyond (1989) [12,13]	Guiding opinions on initiating the long-term care insurance pilots (2016) [1]; Guiding opinions on expanding the long-term care insurance pilots (2020) [2]	Book XI of the Social Code (1994) [7]; Strengthening long-term care acts (Pflegestärkungsgesetze I–III) (2015–17) [7]	The Long-term Care Insurance Act (Kaigo Hoken enacted in 1997, implemented in 2000 and revised in 2017) [8]	Act on Long-Term Care Insurance for Senior Citizens (2007) [14]	Long-Term Care Insurance Model Act (1986) [15]
**Administration**	Government [3,16]	Government [4,16]	Government [6,7,16]	Government [8,9,16,17]	Government [10,14,16,18]	Private insurance: joint administration by private insurers, state regulators, etc. (e.g. National Association of Insurance Commissioners) [19]; public insurance program (mainly Medicare and Medicaid): government administration
**Beneficiaries**	Older people, people with physical disabilities, frailty, and sensory impairment, people with learning disabilities, people with mental health problems, people who misuse substances, and other vulnerable people [3]	Different cities make different determinations based on, for example, disability level (e.g. using the Barthel score or government-specified criteria) and disease (e.g. dementia) [4,16]	People of all ages can apply; Medical Review Boards evaluate if the applicants are ‘in need of care’ and place them into one of five grades (or deny care) [7]	The beneficiaries are divided into two categories: primary insured persons are older adults aged ≥65 y who need LTC; secondary insured persons are adults 40–64 y old who need medical care or assistance with specific chronic diseases [9]	All Koreans, but it mainly targets older people (≥65 y), as well as younger people (<65 y) with age-related LTC needs recognised by LTCI (e.g. dementia or cerebrovascular disease) [10]	Private LTCI: all those who need LTC and are able to pay premiums; Medicare: older adults (≥65 y) and individuals <65 y with long-term disabilities or end-stage renal disease; Medicaid: special groups such as low-income families, the blind, disabled people, and children [16]
**The main source of financing**	Government finance (e.g. central health budgets and local social care budgets) [3,16]	Government finance; individual and employer contributions [4,16]	Government finance; individual and employer contributions [6,7,16]	Government finance contributes 50% (among this, 25% is from the central government, 12.5% from the prefecture level, and 12.5% from the municipality); individuals and employers contribute 50% (among this, 23% is from the primary insured (in principle, withheld from pension benefits), and 27% is from the secondary insured (in principle, half the premium is paid by employers for members of health insurance associations)) [9]	Government finance; individual and employer contributions [10,14,16,18]	Private LTCI: premiums paid by individuals and employers; Medicare: general federal taxes, a mandatory payroll tax for hospital insurance, and individual premiums; Medicaid: government finance at the federal and state levels [16]
**Individual contribution rate**	Not applicable	Fixed amount or calculated based on average salaries [4,16]	For those with children, the contribution rate is about 3.05% of monthly gross income (up to an annually defined threshold) payable by employees and employers (at equal shares in most states, that is, 1.525% each); people aged >23 y without children pay a 0.25% point increased contribution rate (a total contribution of 3.3%); pensioners have to contribute the entire 3.05% [6,7]	For the primary insured, premiums are set by municipalities (they vary geographically depending on local spending), and the premium rates are revised every three years to maintain fiscal balance based on each municipality’s cost projections; for the secondary insured, the contribution is about 0.9% of monthly income [17]	The rate was 0.68% of income in 2020 [18]	Not applicable
**LTC providers**	Public organisations, the private sector (for-profit and non-profit organisations) [3,16]	Public organisations, the private sector (for-profit and non-profit organisations) [4,16]	Public organisations, the private sector (for-profit and non-profit organisations) [6,7,16]	Public organisations, the private sector (for-profit and non-profit organisations) [8,9,16,17]	Public organisations, the private sector (for-profit and non-profit organisations) [10,14,16,18]	Public organisations, the private sector (for-profit and non-profit organisations) [11,16,19]
**LTC payers**	National Health Service, Primary Care Trusts [3,20]; a mixture of public and private funds (e.g. Better Care Fund) [3]; individuals	Government; UEBMI or URRBMI pooled funds [21]; individuals	States, LTCI, private long-term insurers, and individuals [6,7,16]	LTCI (70–90%); individuals (10–30%) [9]	Government, LTCI, National Health Insurance, individuals [10,14,16,18]	Government (Medicare and Medicaid), private LTCI, and individuals [11,16,19]
**Program’s performance**	About 135 000 people were receiving LTC at the end of 2018 [16]	Some cities cover UEBMI and URRBMI enrolees, while other cities only cover UEBMI enrolees; participation is voluntary, and eligibility is stringent [4,16]	In 2018, 72.8 million individuals (87.7% of the population) were covered by mandatory social LTCI, and about 9.2 million individuals (11.1% of the population) were covered by mandatory private LTCI [6]	In 2019, the LTCI system supported 6.28 million service users [9]	In 2018, the number of LTCI beneficiaries was 648 792 [10,18]	There is no universal coverage for LTC services [11,16,19]

LTC – long-term care, LTCI – long-term care insurance, UEBMI – urban employees basic medical insurance, URRBMI – urban and rural residents basic medical insurance

HCBS represent the most commonly offered form of LTC services among countries with established LTCI systems. Institutional care is sometimes offered as a supplement, although intentional ‘de-institutionalisation’ of LTC is increasingly prominent [20,21]. Many LTC beneficiaries prefer to remain at home or in a home-like environment as they age because it benefits both physical care and emotional support. In addition, compared to institutional care, HCBS typically have lower costs, which helps reduce government expenditure and the financial burden of LTC on families and individuals. Therefore, governments in various countries have provided policy support to those who choose HCBS, gradually making HCBS the mainstream LTC service supply method. Meanwhile, LTC services are offered by providers with various ownership structures, including public, for-profit, and non-profit organisations (**Table 1**).

All the examined countries have adopted the ‘apply and evaluate’ method to draw boundaries between participants and beneficiaries. Under this approach, LTCI participants can apply for benefits when they have LTC needs, and the government evaluates whether applicants meet the requirements. Although the specific participant and beneficiary groups under LTCI vary across countries, LTCI’s main target populations are disabled people and older adults who need LTC services (**Table 1**).

Funding for LTCI mainly comes from three channels – government finance, employer payment, and employee payment. Although governments have different levels of responsibility under different models, all the governments in the examined countries have provided funding to some degree. Government responsibility for LTCI financing is largest in countries with the welfare state model, followed by those with the social insurance model, and finally, those with the commercial insurance model (**Table 1**).

While the LTCI systems in the studied high-income countries are more comprehensive than what currently exists in China, they are generally at early development stages. They are not as mature as these countries’ medical insurance systems. Since economic and cultural conditions differ among the examined countries, the specific LTCI implementation measures undertaken also vary. As China is currently in the initial stage of LTCI system design, it should seriously consider the national context (i.e. economic and cultural conditions). In particular, China can draw on its relatively high gross contribution rate for all social security schemes and its Housing Provident Fund, which, among other things, facilitates saving for housing expenses during retirement. Most scholars argue that China should adopt the social insurance model [4,22,23]. The welfare state model would impose a comparatively heavy financial burden on central and local governments since China has a large population compared to the UK. At the same time, the commercial model might not successfully achieve universal coverage since China is a developing country and many people earn only moderate incomes. Indeed, the policies China has implemented to date point toward social insurance [20,22].

## CURRENT CHALLENGES OF LTC IN CHINA

China faces multiple challenges regarding LTC, including increasing demand for care due to population ageing and an insufficient supply of LTC services due to inadequate funding channels. In this context, China needs to construct and implement a robust LTCI system as soon as possible to cope with the advent of rapid ageing.

### Demand for LTC has increased sharply

Demand for LTC services in China has risen and continues to rise sharply for several reasons. First, population ageing is accelerating. According to China’s seventh National Census, the country is home to 264 million adults aged ≥60 years, accounting for 18.7% of the total population. 190.64 million Chinese residents are ≥65 years old, accounting for 13.5% of the total population [24]. From 2000 to 2020, the proportion of older adults increased by 8.4 percentage points; most of this increase occurred between the sixth National Census in 2010 and the seventh National Census in 2020, with the older adult share rising 5.4 percentage points during that timeframe [24].

Second, the number of disabled people, especially disabled older adults, is increasing year by year. 109 million people were estimated to be living with disability in China in 2020, and this number is projected to increase to 136 million by 2030 [25]. In particular, disabled older adults are projected to account for over 57% of all disabled persons by 2030, and this proportion is projected to further increase to over 70% by 2050 if no prevention and control measures are implemented [25]. This increase in the disabled population naturally precipitates a parallel increase in the demand for LTC services [26,27]. In 2015, 15.3% of older adults in China self-reported needing care services, a substantial increase from 13.7% in 2010 and 6.6% in 2000. This demonstrates the urgent demand for LTC services among older adults [26]. Meanwhile, the number of people suffering from chronic diseases is also increasing. In 2019, chronic diseases accounted for 88.5% of total deaths in China; among them, cardiovascular and cerebrovascular diseases, cancer, and chronic respiratory diseases accounted for 80.7% [28].

Third, adult offspring of older adults requiring LTC are likely to turn to LTCI with increasing frequency, and demand for LTC services will correspondingly continue to grow. In China, family members usually assume most of the care responsibilities for older adults, including physical, psychological, and financial aspects [29–33]. However, due to the one-child policy implemented over roughly 30 years (from the 1980s to the 2010s), a single adult offspring will have to care for two older adult parents in many families. This is liable to result in substantial negative effects on the mental and physical health, employment, and income of the adult offspring of older adults [34,35]. Turning to LTCI and professional LTC services represents a natural behavioural response to this challenge.

### The supply of LTC is developing slowly

Just as there are several explanations for the growing demand for LTC services in China, several factors contribute to the insufficient and slowly developing supply of these services. First, China’s current approach to LTCI financing is insufficient and unsustainable, and establishing a diversified portfolio of financing mechanisms merits consideration. At present, the main source of financing for LTCI in most pilot cities is medical insurance funds (accounting for about 70% of LTCI resources), with personal account transfers or payments (about 20%) and public finance (about 10%) accounting for smaller shares [36]. The heavy reliance on medical insurance during the early stages of LTCI piloting is due to the absence of dedicated funding streams for LTCI in many cities, as well as the lack of established independent LTCI funds. However, the limited availability of medical insurance funds might curtail future development of LTCI systems.

Second, the development of HCBS for semi-disabled older adults is slow, and the institutional LTC sector for fully disabled adults is not operating well. China’s central government has set targets for a so-called ‘90-7-3 structure’ of LTC services, under which 90% of older people receive care in their homes, 7% are supported by community-based services, and 3% receive institutional care [4]. However, HCBS in China are currently underdeveloped and concentrated in urban areas, despite the stated desire to have the majority of older adults receive care at home. With regards to institutional care services, the total number of residential care beds in China more than tripled (rising from 2.3 to 7.3 million) from 2008 to 2018, and the number of beds per 1000 people aged ≥65 years more than doubled (rising from 21.4 to 43.6) [4]. However, a substantial number of beds are not occupied, as the average occupancy rate of institutional care beds nationwide is 60%. In addition, about 80% of the older adults who occupy beds can care for themselves. Based on these figures, the overall effective utilisation rate of institutional care beds nationwide is around 12% (20–60%) [37].

Third, there is an inadequate supply of LTC workers, and the professional level of most of these workers and their institutions is insufficient to provide satisfactory care. China has an estimated 300 000 registered long-term care workers nationwide [4], many fewer than Germany (n = 974 138), Japan (n = 2 411 446), Korea (n = 332 332), and USA (n = 2 807 279) [38]. Furthermore, many LTC workers have not received professional training [39]. In October 2020, five government departments, including the Ministry of Human Resources and Social Security and the Ministry of Civil Affairs, jointly issued the Notice on the Implementation of Healthcare Vocational Skills Training Program, which proposed to train more than two million LTC workers between 2020–22. However, such programming still falls short of what is required to meet the demand for qualified LTC workers [36].

## RECOMMENDATIONS

Based on the previous discussion, we propose that China take several steps to strengthen its LTCI system. First, China should issue a unified policy framework to clarify the LTCI system’s principles and develop institutional arrangements informed by the pilot program. China should develop a unified policy document to regulate the scope of LTCI coverage, fundraising channels, contribution groups, contribution rates, and fund management, among other factors, to ensure consistency across regional programs. The central government should also establish a unified standard for assessing LTC needs and the quality and pricing of individual care services to promote the development of the LTC and LTCI systems.

Second, China should consider multiple pathways to establish a stable financing mechanism for LTCI. It could draw on the experiences of other countries and establish a financing mechanism based on employee and employer contributions. Alternatively, China could consider adopting a mixed financing method in which the government, employers, and employees share responsibility. For example, the central and local governments could finance 20–40% of operating funds, with the remaining portion funded through joint contributions from employers and employees. The central and local governments could also share the funding burden through additional government support for low-income and zero-income earners who are unable to contribute [20].

Third, involving a range of provider types could facilitate the smooth implementation of the LTCI system. Government management departments should enhance the utilisation of social and market forces by transitioning from the past ‘direct service provision’ framework to a new ‘social services purchase’ framework to encourage for-profit and non-profit organisations to participate in LTC and LTCI [40]. In addition, to promote HCBS, ensure a sufficient supply of LTC workers, and guarantee a high quality of LTC service provision, private firms could be tasked with leading training programs for LTC workers [41].

## CONCLUSIONS

China needs to actively respond to a series of challenges brought about by imminent population ageing. LTCI not only facilitates quality health care for older adults but also addresses the problem of dwindling labour supply, reduces medical expenses, and improves overall social well-being. In the next stage of the LTCI rollout, the government should focus on building an LTCI system that guarantees basic services (including medical and elderly care), wide-coverage, sustainability, and urban-rural coordination. China should also establish nationally uniform coverage, set contribution rates at reasonable levels, increase HCBS provision and financial investment, encourage different kinds of providers to participate in LTC, accelerate the synergistic operation of LTCI with social security, old-age care services, and health support systems, and promote a fairer and more sustainable development of LTCI.

## References

[1] Ministry of Human Resources and Social Security of the People’s Republic of China. Guiding opinions on initiating the long-term care insurance pilots. 2016. Available: http://www.mohrss.gov.cn/xxgk2020/fdzdgknr/zlbmxgwj/ylbx/201607/t20160705_242951.html. Accessed: 15 November 2020.

[2] National Healthcare Security Administration, Ministry of Finance of the People’s Republic of China. Guiding opinions on expanding the long-term care insurance pilots. 2020. Available: http://www.nhsa.gov.cn/art/2020/9/16/art_37_3586.html. Accessed: 15 November 2020.

[3] Anderson M Pitchforth E Edwards N Alderwick H McGuire A Mossialos E The United Kingdom: Health system review. Health Syst Transit. 2022;24:1–194. 35579557

[4] Feng Z Glinskaya E Chen H Sen G Qiu Y Xu J Long-term care system for older adults in China: policy landscape, challenges, and future prospects. Lancet. 2020;396:1362–72. 10.1016/S0140-6736(20)32136-X 34338215

[5] Chen S Li L Yang J Jiao L Golden T Wang Z The impact of long-term care insurance in China on beneficiaries and caregivers: A systematic review. J Glob Health Econ Policy. 2021;1:e2021014. 10.52872/001c.29559 35083471 PMC8788994

[6] Blümel M Spranger A Achstetter K Maresso A Busse R Germany: Health system review. Health Syst Transit. 2020;22:1–272. 34232120

[7] Organization for Economic Co-operation and Development, European Observatory on Health Systems Policies. Germany: Country Health Profile 2019. Paris, France: Organization for Economic Co-operation and Development, European Observatory on Health Systems Policies; 2019. Available: https://www.oecd-ilibrary.org/content/publication/36e21650-en. Accessed: 3 October 2021.

[8] Organization for Economic Co-operation and Development. OECD Reviews of Public Health: Japan: A Healthier Tomorrow. Paris, France: Organization for Economic Co-operation and Development, European Observatory on Health Systems Policies; 2019. Available: https://www.oecd.org/en/publications/oecd-reviews-of-public-health-japan_9789264311602-en.html. Accessed: 3 October 2021.

[9] Ministry of Health Labour and Welfare. Long-Term Care Insurance System. 2019. Available: https://www.mhlw.go.jp/content/12300000/000614772.pdf. Accessed: 7 August 2022.

[10] Organization for Economic Co-operation and Development. OECD Reviews of Public Health: Korea. Paris, France: Organization for Economic Co-operation and Development; 2020. Available: https://www.oecd-ilibrary.org/content/publication/be2b7063-en. Accessed: 3 October 2021.

[11] Rice T Rosenau P Unruh LY Barnes AJ van Ginneken E United States of America: Health system review. Health Syst Transit. 2013;15:1–431. 24025796

[12] Langan M Community care in the 1990s: the community care White Paper: ‘Caring for People’. Crit Soc Policy. 1990;10:58–70. 10.1177/026101839001002904

[13] Datta S Out-of-pocket costs for long-term care. Ageing Int. 1993;20:55–9. 10.1007/BF03032499

[14] Maags C Long-Term Care Insurance Adoption in East Asia: Politics, Ideas, and Institutions. Polit Policy. 2020;48:69–106. 10.1111/polp.12339

[15] Chen YP Funding long-term care in the United States: The role of private insurance. Geneva Pap Risk Insur Issues Pract. 2001;26:656–66. 10.1111/1468-0440.00146

[16] Tikkanen R, Osborn R, Mossialos E, Djordjevic A, Wharton G. International profiles of health care systems. USA: The Commonwealth Fund; 2020. Available: https://www.commonwealthfund.org/sites/default/files/2020-12/International_Profiles_of_Health_Care_Systems_Dec2020.pdf. Accessed: 3 October 2021.

[17] Rhee JC Done N Anderson GF Considering long-term care insurance for middle-income countries: comparing South Korea with Japan and Germany. Health Policy. 2015;119:1319–29. 10.1016/j.healthpol.2015.06.001 26117093

[18] Kim H Kwon S A decade of public long-term care insurance in South Korea: Policy lessons for aging countries. Health Policy. 2021;125:22–6. 10.1016/j.healthpol.2020.11.003 33189411

[19] Lin H Prince J The impact of the partnership long-term care insurance program on private coverage. J Health Econ. 2013;32:1205–13. 10.1016/j.jhealeco.2013.09.010 24189449

[20] Zhou J Wei Y Ding L The International Comparison and Enlightenment of the Long-term Care Model for the Elderly. Social Security Studies. 2018;3:92–101.

[21] Organization for Economic Co-operation and Development. Long-term Care and Health Care Insurance in OECD and Other Countries. Paris, France: Organization for Economic Co-operation and Development; 2020. Available: https://www.oecd-ilibrary.org/finance-and-investment/long-term-care-and-health-care-insurance-in-oecd-and-other-countries_3eabc286-en. Accessed: 10 March 2022.

[22] Tian Y The Study on the Financial Burden Capacity of Long-term Care Insurance in China – A Discussion on the Rationality of Long-term Care Insurance System Based on Medical Insurance. Social Security Studies. 2020;1:33–47.

[23] Chen S Li L Jiao L Wang C Long-term care insurance and the future of healthy aging in China. Nat Aging. 2023;3:1465–8. 10.1038/s43587-023-00540-9 38093139

[24] National Bureau of Statistics of China. Interpretation of the Seventh National Census Communique. 2021. Available: https://www.stats.gov.cn/sj/sjjd/202302/t20230202_1896484.html. Accessed: 3 October 2021.

[25] Luo Y Su B Zheng X Trends and challenges for population and health during population aging – China, 2015-2050. China CDC Wkly. 2021;3:593–8. 10.46234/ccdcw2021.158 34594944 PMC8393078

[26] China Research Center on Aging, National Commission for Population Aging of China. The fourth sampling survey on the living conditions of the elderly population in urban and rural China. 2021. Available: http://www.crca.cn/index.php/19-life/27-2015.html. Accessed: 3 October 2021.

[27] Jing L Chen R Jing L Qiao Y Lou J Jing X Development and enrolee satisfaction with basic medical insurance in China: A systematic review and stratified cluster sampling survey. Int J Health Plann Manage. 2017;32:285–98. 10.1002/hpm.2430 28664591

[28] National Health Commission of the People’s Republic of China. Report on Nutrition and Chronic Disease Status of Chinese Residents. 2020. Available: http://www.scio.gov.cn/xwfb/gwyxwbgsxwfbh/wqfbh_2284/2020n_4408/2020n12y23rsw/. Accessed: 3 October 2021.

[29] Colombo F, Llena-Nozal A, Mercier J, Tjadens F. Help Wanted?: Providing and Paying for Long-Term Care. Paris: Organization for Economic Cooperation and Development; 2011. Available: https://www.oecd-ilibrary.org/content/publication/9789264097759-en. Accessed: 3 October 2021.

[30] Scheil-Adlung X. Long-term care protection for older persons: A review of coverage deficits in 46 countries. Geneva: International Labour Office; 2015. Available: https://www.ilo.org/publications/long-term-care-ltc-protection-older-persons-review-coverage-deficits-46. Accessed: 18 August 2024.

[31] Giles J Wang D Zhao C Can China’s Rural Elderly Count on Support from Adult Children? Implications of Rural-to-Urban Migration. J Popul Ageing. 2010;3:183. 10.1007/s12062-011-9036-6

[32] Van Houtven CH Norton EC Informal care and health care use of older adults. J Health Econ. 2004;23:1159–80. 10.1016/j.jhealeco.2004.04.008 15556241

[33] Pickard L Wittenberg R Comas-Herrera A King D Malley J Care by Spouses, Care by Children: Projections of Informal Care for Older People in England to 2031. Soc Policy Soc. 2007;6:353–66. 10.1017/S1474746407003685

[34] Rubin RM White-Means SI Informal Caregiving: Dilemmas of Sandwiched Caregivers. J Fam Econ Issues. 2009;30:252–67. 10.1007/s10834-009-9155-x

[35] Heitmueller A The chicken or the egg?: Endogeneity in labour market participation of informal carers in England. J Health Econ. 2007;26:536–59. 10.1016/j.jhealeco.2006.10.005 17098311

[36] Sun J Experience, Problems and Policy Suggestions of the Pilot Long-term Care Insurance in China. Price. Theory Pract. 2021:1–6.

[37] Wangyi News Report. Market Status of China’s Nursing Homes Industry: the vacancy rate of nursing homes is as high as 50%. 2021. Available: https://www.163.com/dy/article/GLN0P54F0514HA3H.html. Accessed: 10 March 2022.

[38] Organization for Economic Cooperation and Development. Long-Term Care Resources and Utilisation, Nurses and Caring Personnel. 2019. Available: https://data-explorer.oecd.org/vis?fs[0]=Topic%2C1%7CHealth%23HEA%23%7CHealthcare%20resources%20and%20equipment%23HEA_RES%23&pg=0&fc=Topic&bp=true&snb=20&vw=ov&df[ds]=dsDisseminateFinalDMZ&df[id]=DSD_HEALTH_REAC_EMP%40DF_NURSE&df[ag]=OECD.ELS.HD&df[vs]=1.0&dq=.10P3HB...MINU.P.&pd=2015%2C&to[TIME_PERIOD]=false&ly[cl]=TIME_PERIOD&ly[rw]=REF_AREA. Accessed: 13 June 2022.

[39] Wang L Tian Z Problems in China’s Long-term Care Insurance System and Suggestions for Improvement. Social Governance Review. 2021;4:59–65.

[40] Wang Q Zhou Y Ding X Ying X Demand for Long-Term Care Insurance in China. Int J Environ Res Public Health. 2017;15:6. 10.3390/ijerph15010006 29271906 PMC5800106

[41] Ling W Dong Y Demand Analysis, International Experience and Chinese Program for Long-term Care-A Literature Review. Social Security Studies. 2019;4:105–11.

